# European funds and firm performance: evidence from a natural experiment

**DOI:** 10.1007/s11187-025-01052-z

**Published:** 2025-06-02

**Authors:** José Mesquita, João Pereira dos Santos, José Tavares

**Affiliations:** 1 Nova School of Business and Economics, Universidade Nova de Lisboa, Campus de Carcavelos, Lisbon, Portugal; 2 Queen Mary University of London, London, UK; 3 ISEG – University of Lisbon, REM/UECE, Lisbon, Portugal; 4 IZA – Institute of Labor Economics, Bonn, Germany; 5 Centre for Economic Policy Research (CEPR), London, UK

**Keywords:** Grants, Regional policy, Private firm, Municipalities, Portugal, R10, R30, H25

## Abstract

This paper examines the impact of European Union (EU) funds on the performance of private firms. We exploit a quasi-natural experiment arising from an administrative redrawing of geographical boundaries, which led to a discrete change in regional eligibility. This caused a sudden and substantial increase in access to EU grants directed at firms located in 33 Portuguese municipalities. Using a comprehensive linked employer-employee administrative dataset which covers the universe of private firms between 2003 and 2010, our difference-in-differences estimates uncover a significant and positive causal effect of increased eligibility on firms’ sales, labor productivity, and average wages, while employment is not significantly altered. Although firms’ sales in the non-tradable sectors are positively impacted, firms’ sales in more competitive, tradable, sectors remain unaffected by increased access to EU funds.

## Abstract (plain language summary)

Splitting a region to increase EU funding eligibility boosts firm sales in the short-run, but only for less competitive, non-tradable, sectors. Increased eligibility did not affect employment or firm dynamics, while average wages rose slightly. This study examines the effects of increased eligibility to EU funds on firm performance in Portugal, following the administrative redrawing of geographical units. By splitting Lisbon into two areas, the poorer half improved their access to EU funding. Firms in this region saw an average sales boost of 7.4%, but the impact was limited to firms in sectors shielded from international competition. Employment and firm creation remained unchanged, while average wages marginally increased. Larger firms used the extra funds to hire top managers, driving up wage disparity. Our findings suggest that EU funds benefit certain sectors more than others, emphasizing the importance of targeted funding policies. These results highlight the need for refined place-based policies that consider firm and sector heterogeneity to maximize the effectiveness of EU funding.

## Introduction

As stated in the Treaty of Lisbon, the European Union (EU) promotes socioeconomic cohesion among its member states, in part by directing substantial budgetary transfers to less developed regions with income per capita below 75% of the EU average. The stated objective is to boost regional income, employment growth, and support business creation. Thus far, the actual results of this policy are hard to assess, suggesting a clear need for micro-based causal empirical evaluations. The empirical evidence indicates that, *on average*, transfers appear to have been effective in promoting regional growth and lowering regional disparities (Becker et al., 2010; Pellegrini et al., 2013; Giua, 2016). Nonetheless, outcomes vary considerably depending on local conditions (Becker et al., 2013), and effects may be subject to diminishing returns (Becker et al., 2012; Cerqua & Pellegrini, 2018), or prove temporary (Barone et al., 2016; Di Cataldo, 2017; Becker et al., 2018). Notably, GDP per capita across EU- 15 metro regions has diverged since the mid- 2000 s (Ehrlich & Overman, 2020).

In this paper, we exploit a quasi-natural experiment consisting of the redistricting of the Lisbon NUTS 2 area (Portugal), splitting it in two distinct areas, leading to a sudden increase in eligibility to EU funds in specific regions.^1^ This decision, made by the Portuguese Government and approved by EU institutions, came in the wake of the Lisbon area surpassing the 75% of EU average income per capita threshold, which compromised the future flow of funds. By separating the region, the poorer, more peripheral areas regained eligibility status, a strategy that over the last decades has been employed by several EU governments, precisely to maximize their eligibility for EU funds.^2^

EU regions tend to lose, rather than gain eligibility in the growth process. Thus, and unlike most empirical papers thus far, we analyze the impact of *increased* eligibility on firm performance.^3^ We further contribute to the literature by using a comprehensive linked employer-employee administrative dataset that covers the universe of Portuguese private firms, between 2003 and 2010, allowing us to uncover how firms react to potential new funding opportunities. We further consider heterogeneous effects that we believe key to understand the mechanism through which EU grants affect firm performance.

Overall, we find evidence that a higher eligibility status to EU funds for a municipality increases private firms’ sales, *on average*, by 7.4% *vis-à-vis* firms in municipalities experiencing no change. While average monthly wages increase marginally, particularly for newly hired workers, total employment numbers do not seem to change in response to the shock. We uncover a null impact on the total number of firms and firms’ creation in Treated municipalities, as well as no change in the probability of firm closure, indicating increased access to EU funds does not significantly affect firms’ dynamics.

Heterogeneity analysis reveals that positive effects are concentrated in firms in the less competitive, 

Non-Tradable, sectors. In other words, the performance of firms subject to fiercer international competition remains unaltered after the treatment. When we investigate how firms allocate the increase in EU funds, we find that non-micro firms used these new resources to hire top managers and, as a result, average wages and wage disparity within each firm increased. On the other hand, the management structure in micro-firms remained unaltered, suggesting that these investments become significantly more important as firms get larger (Caliendo et al., 2020).

Our intent-to-treat estimates from a difference-in-differences specification (hereafter, diff-in-diff) yield the causal impact of increased eligibility under the parallel trends assumption—had the redistricting not been implemented, and the outcomes of Treated firms would have evolved similarly to those of comparison firms. For that reason, our control group only considers firms that initially were and later remained in high-eligibility status regions. In addition, we exclude from the control group any municipalities that neighbor the treated municipalities to mitigate possible spillover effects. When we consider possible spillover effects from Treated to neighboring areas, we show that the latter do not witness significant changes *vis-à-vis* comparison municipalities. We present comforting evidence that the parallel trend assumption is likely to be supported in this context both by descriptive graphical inspection and event study specifications. Moreover, our findings are robust to time and the spatial sensitivity checks, including different control groups and the exclusion of the 2009–2010 crisis period.

Using administrative balance sheet data, we also examine firms’ financial structure and find no changes in assets, liabilities, equity, or business profit share. This suggests that increased eligibility did not substantially affect how firms finance themselves.

We complement our findings with municipality-level data to assess the effects on geographic exposure to the policy. We find that European funds transferred to firms in Treated areas increase substantially, but there is no evidence of an increase in EU transfers to local government. Furthermore, there is no evidence of a change in the amount of transfers from the central government to the treated areas, and also no change in current local government expenditures. These concurring facts suggest the improvement in private firms’ performance is indeed the result of increased eligibility and not the response to other sources of funds. Notably, increased eligibility is associated with an increase in local income, as proxied by electricity consumption for domestic purposes, suggesting that increased firm sales may stem from an increase in individuals’ and workers’ incomes.

Dvouletý et al. (2021) offer an overview of the impact of public investment to support small and medium-sized enterprises (SMEs) in Europe and observe a positive impact of grants on firm survival, employment, and sales, with mixed findings for labor productivity. Most of the studies reviewed use non-supported firms as a control group for supported firms, mitigating concerns with selection into treatment. However, other selection biases may still be present: firms that receive support might be systematically better in both typically observed characteristics such as size, productivity, or financial health, but also in typically unobserved characteristics such as management quality or links with political parties or government. If successful applicants for public support are chosen on the basis of their potential for growth or survival, the outcomes of the support should be attributed to these pre-existing differences, not the policy itself. By comparing firms in different regions after an arguably exogenous policy change taken at the central government level, we are able to overcome most of these caveats.

Important policy implications the design of place-based policies (OECD, 2023) can be drawn from our results. These implications go beyond the Portuguese context as, in the last decades, several EU governments introduced changes to their regional statistical units precisely to maximize their eligibility to EU funds. First, our results point to the importance of relying on firm-level data, as the effects of increased EU eligibility are heterogeneous, with the effect on sales driven solely by firms in Non-Tradable sectors. As emphasized by Dvouletý et al. (2021), firm-heterogeneity must be considered in evaluation studies to better channel public funds to firms that have a higher likelihood to use them more efficiently. Second, the null effects on sales, productivity gains, and employment by firms in Tradable sectors suggest that EU regional funds may sometimes act as a distributional policy, with a relative increased importance of more protected industries from external competition (Benigno & Fornaro, 2014). These lessons are especially important as European regions heretofore strongly supported by the EU’s Cohesion Policy witness an increase in voting for Eurosceptic political parties (Fidrmuc et al., 2019; Rodríguez-Pose & Dijkstra, 2021), an effect that can be mitigated if these investments are coupled with tangible improvements in local conditions (Albanese et al., 2022; Crescenzi et al., 2020).

The remainder of this paper is organized as follows. Section 2 reviews the relevant literature on the effect of regional funds, Sect. 3 presents the estimation methodology and the data, and Sect. 4 the results. Section 5 concludes.

## Related literature

Growth and convergence across European regions have been a political priority of the EU for decades. It gained importance over time as relatively less prosperous countries in Southern and then Eastern Europe adhered.

Hampered by several econometric issues, empirical evidence on the success of EU regional policy is mixed. While Boldrin and Canova (2001) detected no statistically significant effects of EU regional policy on per-capita-income growth of recipient regions, conditional on standard drivers of growth, positive effects on agglomeration and industry location are reported in Midelfart-Knarvik and Overman (2002). This ambiguity may stem from endogeneity issues and requires the use of causal evaluation techniques.^4^

Becker et al. (2010) first exploited the fact that Objective 1 funding (i.e., for lagging regions) is based on a clear assignment rule, with a simple threshold that affects a region’s eligibility: NUTS 2 regions are eligible for funding if their GDP per capita is less than 75% of the EU average. These authors exploited a fuzzy regression discontinuity design (RDD) with data from three programming periods (from 1989 to 2006) to find that, *on average*, Treated regions grow significantly faster than regions just above the 75% threshold.^5^ No effects on employment growth were uncovered. Becker et al. (2012) distinguished average and marginal effects, in which the former may be positive but the latter negative, implying that the optimal funding has been surpassed. Becker et al. (2013) show that regions with high levels of human capital and good institutions were able to use funds more efficiently.

Four more recent papers analyze the impacts of funds for different regions within a single country with regional data. Barone et al. (2016) focused on the post-expiry period to examine the persistence of the economic boost to “convergence” regions after the termination of access to EU Regional Funds. Their findings highlighted that exiting the program hampers regional per-capita GDP growth. Giua (2016) examined municipalities contiguous to the municipalities affected by a policy change to identify the effects of EU Regional Policy in a panel of Italian regions. She finds a positive impact on employment levels. Di Cataldo (2017) estimated the impact of EU funds in Cornwall and South Yorkshire, regions that were among the greatest beneficiaries of EU funds in the UK. Using synthetic control methods, he shows that the income gap across regions has fallen with EU funding, and labor market prospects have improved. Cerqua and Pellegrini (2018) use a regression discontinuity design and conclude that, despite portraying an average positive effect on regional growth, exceeding funds could have been allocated to other lagging Italian regions more efficiently.

We contribute to the literature evaluating cohesion policy funds using the universe of private firms as units of observation, rather than municipalities or NUTS 2 regions. This choice is crucial if we want to better understand how firms make decisions and the mechanisms through which funds affect this process. Fattorini et al. (2020), using propensity score matching techniques and focusing exclusively on manufacturing firms, report a positive effect of EU Regional Funds aimed at investments in R&D on firms’ total factor productivity, particularly among the least efficient. Bondonio and Greenbaum (2006), analyzing firms in Northern and Central Italy’s Objective 2 regions, find a positive impact of EU Regional Funds on employment growth, yet estimating relatively high costs per job created. Another relevant reference is Beņkovskis et al. (2019), who show that, after conditioning on the fact that more productive and larger firms have a higher propensity to acquire EU funds; these regional support programs boost firms’ turnover and employment in Lithuania.

For Portugal, Santos (2019) relies on a sample of around 300 firms that applied for an innovation subsidy granted by European funds during the 2007–2011 period, finding positive effects on employment, sales, and investment for those who received it. Using data for the same time period, Martins (2021) investigates the impact of a large training program sponsored by the European Social Fund (ESF) concluding that it had a significant positive effect on sales, value added, employment, and productivity. Alexandre et al. (2024) examine the impact of being awarded a second investment grant to the same firm, uncovering a positive effect on labor productivity for small firms.

Our paper further relates to the literature on the causal impact of place-based policies, surveyed in Kline and Moretti (2014a). Leveraging on rejected and future applicants to the US Empowerment Zones program as comparison groups, Busso et al. (2013) show that neighborhoods receiving considerable Federal assistance in the form of tax breaks and job subsidies observed an increase in employment and local workers’ real wages. Kline and Moretti (2014b) study the long-run effects of the Tennessee Valley Authority policy using as controls similar institutions proposed but never approved by the US Congress, showing how manufacturing employment increased after federal transfers had fallen.^6^

Place-based policies, such as the EU Structural and Cohesion funds, can possibly deliver effects that go beyond those found in the targeted area (Cerqua & Pellegrini, 2017; Glaeser & Gottlieb, 2009). In this paper, we also consider possible spillover effects by analyzing the effects of treatment on neighboring municipalities. In theory, spillover effects can have either positive or negative effects. If policies are successful at creating new establishments and jobs that would not have emerged in the absence of incentives, there may be a positive effect on surrounding areas through the forces of agglomeration and local multipliers (Moretti, 2010). However, the effects on the neighboring areas may also be negative if spatially targeted policies have business-stealing effects (Andini & Blasio, 2014; Einiö & Overman, 2020; Hanson & Rohlin, 2013).

## Empirical approach

### Institutional background

Portugal has been a recipient of European funding in the context of the distinct Community Support Framework (CSF) phases. Regions whose per capita GDP lies below the threshold of 75% of the European average were eligible for more generous funding than richer areas: according to the so-called Objective 1 (before 2006) or Convergence region funding (after 2007). Differences in regional eligibility imply that more (less) developed regions face a lower (higher) likelihood of having a given project accepted and receive fewer (more) resources from the EU cohesion and structural funds.

The authority to redraw NUTS regions lies in the Central Government of each member state, and any changes have to be communicated to the European Commission and need to be approved by the Eurostat. In 2002, the Portuguese government approved Decree Law 244/2002 which defined the new NUTS 3 configuration that would be adopted in the regulation (EC) of the European Parliament and of the Council (No. 1059/2003). This decision was criticized by several political parties at the time and, as Portugal held unexpected elections in 2005, it remained unclear whether these reforms would still be in place in 2007.^7^

After 2007, Mainland Portugal was divided into three distinct regional groups as far as eligibility for EU funds is concerned, as illustrated in Fig. 3 in the Appendix. The first comprises the North, Centre, and Alentejo regions, which are part of the Convergence objective, associated with the most favorable access to funding. The second is the Algarve region, in the south, part of the phasing out regime, with per capita GDP above the 75% income threshold – for the 25 EU countries considered at the time, but still below the 75% of average income for EU- 15. The third is the smaller NUTS 2 Lisbon region that resulted from the administrative breakup, standing as the only area above the 75% average for EU- 15, and thus part of the Competitive objective, with lower eligibility.^8^

At this time, the EU regional development policies started to move away from investments in flagship high-technology enterprises or large-scale infrastructure projects to encourage local entrepreneurs with funds for the technological upgrading of their processes and training of human capital (McCann & Ortega-Argilés, 2016). One important difference between the CSFs before and after 2007 is therefore related with the thematic allocation of funds: investment in infrastructures saw its relative importance diminished from 48 to 39%, while investment in professional training increased, from 17 to 23% (Pires, 2017).

### Data

In the empirical analysis of this study, we benefit from a longitudinal administratively linked employer-employee dataset, *Quadros de Pessoal*, compiled by the ministry responsible for employment affairs and, for that reason, of mandatory compliance to respond. *Quadros de Pessoal* covers virtually all firms with at least one wage earner in the whole of mainland Portugal.^9^ We retrieved information both at the worker level, including earnings and education, and firm level—sales, number of employees, sector of economic activity, location, and legal structure.^10^

We analyze the period between 2003 and 2010. We do not include the period after 2010 to avoid confounding factors resulting from the effects of the Sovereign Debt Crisis in Portugal. In May 2011, Portugal signed a €78 billion financial assistance program with the European Commission, European Central Bank, and the International Monetary Fund. This agreement imposed a series of stringent austerity measures, including cutting public sector wages by up to 23%, reducing pensions by 15%, and increasing the value-added tax from 21 to 23%, accompanied by a reduction in public expenditure. While successful in stabilizing public finances and restoring investor confidence, Portugal faced a sharp recession, with unemployment peaking at 17.5% in early 2013. This structural break could have had heterogeneous regional effects depending on the pre-crisis demographic and economic conditions.^11^

We selected four firm-level indicators from *Quadros de Pessoal* to evaluate firm performance. Those indicators are total sales—in € per year, the number of total workers, labor productivity—measured as the sum of sales per worker, and monthly average wages—which includes the fixed and the variable wage components. We winsorize these levels at 1% from each tail.^12^

We take the inverse hyperbolic sine (ihs) transformation of the first two dependent variables, an approach that has the advantage of allowing us to consider zeros in variables, even though we show that our results are robust to different transformations, namely logarithmic. Following Bellemare and Wichman (2020), with the inverse hyperbolic sine, the interpretation of marginal effects approximates the natural logarithm of that variable when the untransformed means of such variables are large enough (as a rule of thumb, above 10).

We also test whether treatment has an impact on firm dynamics looking into the probability of exit using a dummy variable that takes value one if the firm closes and zero otherwise. Moreover, we aggregate firm data at the municipal level to examine the effect on the number of total firms and on the number of firms entering the market.

We complement our analysis with firm level and with municipal level administrative data. To assess whether the change in European funds eligibility affected firms’ financial structure, we use annual balance sheet information from S*istema de Contas Integradas das Empresas* (SCIE), available from 2004 onwards.^13^ From this source, we extract information on assets, liabilities, equity, and business profit share. The profit share is defined as gross operating surplus over gross value added at market price, capturing the portion of value added that remunerates capital.

In the municipal level analysis, we rely on several data sources. Micro beneficiary data on European funds at the firm level is not available for the pre-treatment period. However, we were able to obtain data on European funds transferred to firms from the Central Government, namely the Central State Administration, which is the Management Authority for the Competitiveness and Internationalization of European Funds for firms (COMPETE) and to municipalities from the Directorate General of Local Government (DGAL), both aggregated per municipality since 2003. Information on electricity consumption, for domestic and industrial purposes—in thousands of kilowatt hours, is obtained from the government agency for Energy and Geology (DGEG). Data regarding transfers from the central government to municipalities, as well as concerning the current expenses of municipalities (in Euros), are obtained from DGAL.

We display the descriptive statistics for firm-level variables in Tables 6 and 7, while we report descriptive statistics for municipal-level variables in Table 8 of the Appendix. In both cases, we present information for the pre-treatment and post-treatment periods.

### Identification strategy and econometric analysis

Considering that each firm’s likelihood of access to EU funds depends on a range of observed and unobserved variables, a mere comparison between subsidized and non-subsidized firms in a certain region will likely produce biased results. Instead, we rather assess the impact of higher eligibility on firm performance using a natural experiment, a change that is entirely exogenous from the point of view of individual firms, in an intention to treat (ITT) setting. The ITT approach provides an estimate that is often of direct interest to policymakers as it reflects the real-world scenario where not all eligible firms may actually receive treatment (in our case, benefit from increased EU eligibility), due to factors like non-compliance or administrative delays.

We thus exploit the spatial discontinuity in access to European funds which occurred between 2006 and 2007, derived from the redistricting of the Lisbon NUTS 2 area. This change was decided by the central government, with the approval of the European Commission and the Eurostat.^14^ This is important for our identification strategy given that Portuguese firms are small (Cabral, 2007) and with very limited capacity for lobbying. Therefore, from their point of view, changes in the EU funds eligibility are plausibility exogenous. A set of contiguous municipalities to the north of Lisbon were singled out and witnessed a sudden raise in eligibility to EU funds, thus experiencing a break between the pre-treatment period, 2003 to 2006, and the post-treatment period, from 2007 to 2010.

As such, our identification strategy uses as our Treated group the universe of the 39,748 private firms located in the 33 municipalities pertaining to the NUTS 3 regions of Oeste, Médio Tejo, and Lezíria do Tejo, i.e., those who gained greater access to EU funds due to the administrative territorial redrawing.

**Fig. 1:**
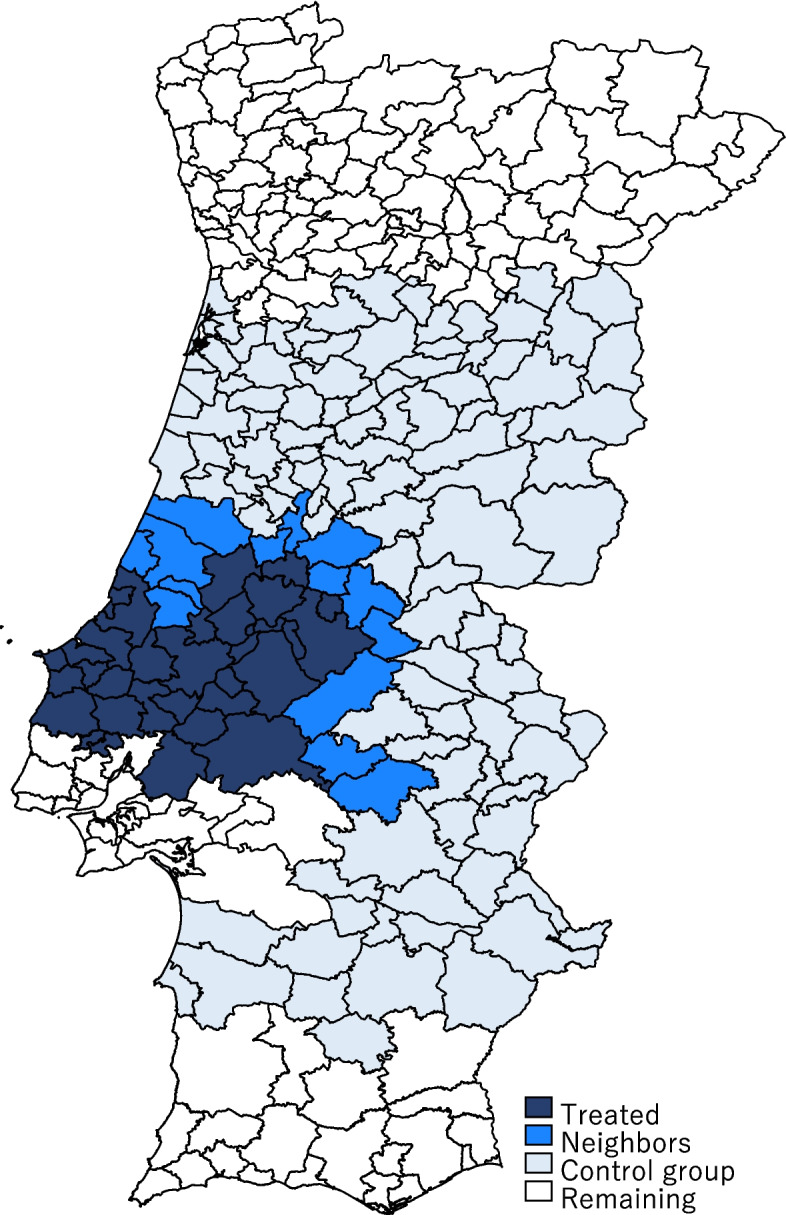
Geographical distribution of the neighbors and comparison municipalities

The comparison group is composed of firms in any of the 104 municipalities pertaining to Centro and Alentejo regions who have experienced no change whatsoever in their or their neighbors’ eligibility for EU Regional Funds. As shown in Fig. 1, we exclude firms neigbouring Treated areas in a “buffer-zone” or “donut-hole” approach to mitigate the possibility of spillover effects from treatment, as it is well-known that methods comparing outcomes in a treated region to those in adjacent regions may yield biased estimates for policies with spillover effects (Jardim et al., 2022). The comparison municipalities have not been subject to any change in eligibility status, therefore, absent possible spillover effects, they are untreated by the redistricting shock. We also investigate this possibility of spillover effects, in Sect. 4.5.

For the vast majority of variables employed in this study, balance tests, presented in Table 9, do not uncover any statistically significant difference between Treated and control groups for 2006, the year prior to the start of the treatment. Nevertheless, as a robustness exercise, we include the more distant NUTS 2 north region in the control group.

Our baseline diff-in-diff regressions estimate the average intent-to-treat effects derived from a standard ordinary least squares (OLS) model as follows:

1 $${Y}_{\text{imt}}=\delta {Post}_{\text{t}}\bullet {Treated}_{\text{m}}+ {\gamma }_{\text{t}}+{\alpha }_{\text{i} }+{e}_{\text{imt}}$$

where ${Y}_{\text{imt}}$ are the outcome variables for a firm $i$, in a municipality $m$, in year $t$. ${Post}_{\text{t}}$ accounts for the treatment period (2007–2010), and ${Treated}_{\text{m}}$ is a binary variable signaling firms producing in municipalities that gained eligibility under *Objective 1/convergence*. ${\gamma }_{\text{t}}$ are year fixed effects, and ${\alpha }_{\text{i}}$ are firm fixed effects—i.e., controls for characteristics of firms that are time-invariant.^15^${e}_{\text{it}}$ accounts for clustered standard errors per NUTS 3, the level of assignment to treatment, as in Bertrand et al. (2004) and Abadie et al. (2023). The parameter of interest is $\delta$, measuring the impact on a firm located in a region whose eligibility to access EU funds increases.

We also implement a difference-in-differences event study design, with several advantages (Roth et al., 2023). First, we can present further evidence suggesting that there are no differential pre-trends between treatment and comparison groups (Roth, 2022). Note that this also mitigates concerns with possible anticipation effects. In the absence of different pre-trends, the identifying assumption is that no systematic factors drive both the shock and the outcomes of interest. Second, the event study makes it possible to evaluate the impact of the shock in the outcome variables in the short and medium run—in this case, up to 4 years. Denoting ${Y}_{\text{imt}}$ as the outcome variable in firm $i$, municipality $m$, and year $t$, the regression model reads as follows:

2 $${Y}_{\text{imt}}={\sum }_{k=2003}^{2005}{\delta }_{\text{k}} {Treated}_{\text{m}}+{\sum }_{k=2007}^{2010}{\delta }_{\text{k}} {Treated}_{\text{m}}+{\gamma }_{\text{t}}+{\alpha }_{\text{i}}+{e}_{\text{imt}}$$

where ${\delta }_{\text{k}}$ is our outcome of interest measuring the year-by-year effect of producing in a Treated region, and the remaining variables are defined as before. The omitted year is 2006, the last year before treatment.

## Firm-level results

### Baseline results

**Fig. 2:**
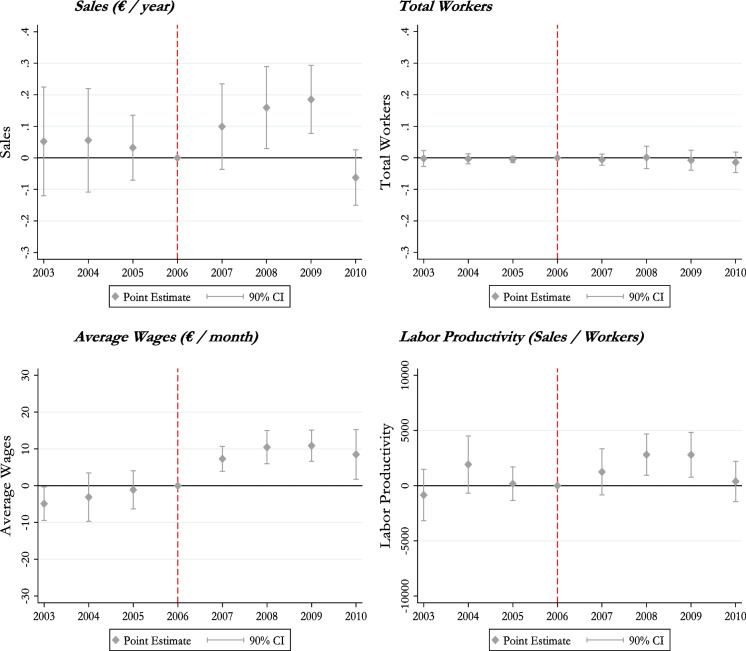
Event studies. Notes: This figure presents the results of Eq. (2), with a confidence interval of 90%. Sales and total workers were transformed using the inverse hyperbolic sine approach

**Fig. 3:**
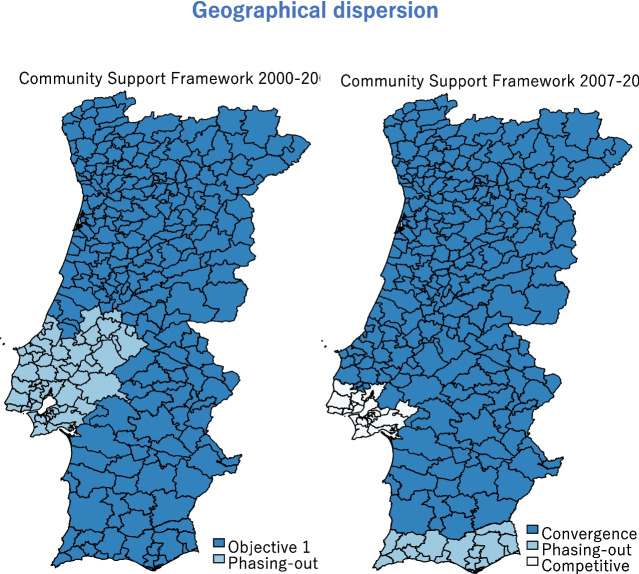
Geographical dispersion. Notes: Firms placed in municipalities with darker colors are more likely to be eligible for EU funds. Firms in Objective 1 (2000–2006) and Convergence (2007–2013) regions are therefore more likely to be eligible

We start by presenting the event study diff-in-diff estimates from computing Eq. (2) in Fig. 2 for the four main dependent variables using 90% confidence intervals. As can be seen for all cases, we find evidence indicating that the parallel trends’ assumption is not rejected in this setting.^16^ We also observe a positive causal effect of the increase in eligibility on sales and labor productivity in 2008 and 2009. The fact that the impact of treatment for 2007 is not statistically significant is consistent with the idea that, in that year, there may still be some spending from the previous funding period and, at the same time, some of the funding from the new period may be slow to start off. In addition, we find that the effect is not persistent and drops to zero in 2010. As to the remaining dependent variables, we find economically small (as in the case of average wages) or non-significant treatment effects.

Our baseline diff-in-diff specification estimates from Eq. (1) are presented in Table 1—panel A and confirm the statistically significant positive impact of the eligibility on firms’ sales (in column 1), corresponding to an increase of about 7.4% *vis-à-vis* firms in comparison municipalities.^17^ We uncover estimates that are statistically indistinguishable from zero for the effect of the treatment on the number of workers (in column 2), suggesting that while Treated firms sell significantly more, this does not create more employment. As for average wages (in column 3), our estimates show a significant increase, albeit of small magnitude, so that producing in a region that gains access is associated, *on average*, with a wage increase of around 11€/month, or about 2% of the average value of monthly average wages in the treatment and control groups. We further separate the effects for new hires (in column 3.1) and for incumbent workers (in column 3.2), as in the short-run wage adjustments are likelier to be noticed for workers with a higher labor mobility. In fact, we observe that the increase in eligibility benefitted relatively more workers entering new firms *vis-à-vis* incumbent workers, as we estimate an increase of around 18€/month in wages for the former group, in comparison with 11€/month for the latter. We also find a significant rise in labor productivity (in column 4).

**Table 1:** Diff-in-diff baseline results (panel A) and sectoral analysis (panel B)

	Sales (ihs)	Total workers (ihs)	Average wages	Average wages—new hires	Average wages—incumbent workers	Labor productivity
	(1)	(2)	(3)	(3.1)	(3.2)	(4)
Panel A: full sample						
Treated × post-treatment	0.074*	− 0.003	11.193***	18.151**	11.188***	1575.692**
	(0.04)	(0.02)	(2.42)	(7.28)	(2.40)	(704.46)
Adj *R*^2^	0.36	0.88	0.73	0.48	0.73	0.71
*N*	451,318	451,442	451,442	75,741	359,578	451,317
Panel B: by sector – non-tradable versus tradable						
Non-tradable						
Treated × post-treatment	0.094**	− 0.004	11.334***	22.877*	11.995***	2108.291**
	(0.04)	(0.02)	(3.20)	(11.84)	(2.84)	(831.16)
Adj *R*^2^	0.36	0.87	0.73	0.46	0.73	0.74
*N*	297,737	297,811	297,811	44,360	235,007	297,736
Tradable						
Treated × post-treatment	0.022	− 0.001	10.695***	13.509	9.190**	− 124.995
	(0.06)	(0.02)	(3.03)	(15.09)	(3.12)	(940.35)
Adj *R*^2^	0.38	0.90	0.73	0.48	0.74	0.64
*N*	151,226	151,274	151,274	30,290	122,767	151,226
Year fixed effects	Yes	Yes	Yes	Yes	Yes	Yes
Firm fixed effects	Yes	Yes	Yes	Yes	Yes	Yes

Dependent variables in columns (1) and (2) were transformed using the inverse hyperbolic sine approach (ihs). Our regressor of interest, treated × post-treatment, indicates firms producing in one of the 33 Treated municipalities during the treatment period (2007–2010). Our analysis includes the 2003–2010 period. Owing to data limitations, namely the fact that we are unable to track some workers when they change firms, the analysis of average wages for new hires (column 3.1) and incumbent hires (column 3.2) does not cover the entire sample; furthermore, new firms are not included in these columns. Clustered standard errors, at the NUT3 level, are presented in parenthesis. Significance level at which the null hypothesis is rejected: *** 1%, ** 5%, * 10%

As discussed in Sect. 2, most of the studies on the impact of public SME grants on firm performance in the EU use non-supported firms that applied for grants as a control group for supported firms. By relying on a quasi-natural experiment and comparing firms in different regions exploiting an arguably exogenous policy change taken at the central government level, our methodology takes a different approach. For this reason, our estimates complement these previous studies.

Our point estimates on the effects on sales are in line with results from the existing literature.^18^ Furthermore, while less commonly examined in other studies, our point estimates on the effects of average wages of public grants are also comparable with the previous literature.^19^ The pertinent literature, however, usually uncovers positive effects from public grants on the workforce in supported firms.^20^ This is not the case in our setting, as we estimate a null effect on employment. One possible explanation for these patterns can be that employment estimates found in the literature may suffer from spillover and/or general equilibrium concerns, where subsidized firms use these resources to attract workers from non-subsidized firms (Cerqua & Pellegrini, 2017), thereby overstating the true effects.

We next turn our attention to the possibility that EU regional funds may spur sales and have purely distributional effects from tradable to non-tradable sectors, without any real effects on firm performance through productivity and efficiency.

### Non-tradable vs. tradable sectors

In Table 1—panel B, we find that the impetus behind the sales and the labor productivity increase is driven solely by the Non-Tradable sector, with a statistically significant increase in sales for firms in this sector of more than 9%, on average.^21^ There is no effect whatsoever for both indicators on the Tradable sector, i.e., for firms competing in the international markets, suggesting that increased access to EU regional funds does not promote a more efficient entrepreneurial context, rather it increases sales by firms sheltered from competition (as proxied by their sector of activity). The monthly average wages increase relatively uniformly across sectors—11€/month. In what regards to employment, we find no evidence of a significant effect in both sectors.

Our results are consistent with the insights of the theoretical model of the financial resource curse (events of large capital inflows from abroad coupled with stagnant growth) for a two-sector (tradable and non-tradable) small open economy presented by Benigno and Fornaro (2014). The authors argue that periods of abundant access to foreign capital shift resources from the traded sector, which is the source of endogenous productivity gains, to the nontraded sector. This allocation of resources limits the development of a dynamic export sector and hinders medium- to long-run competitiveness. Although there is evidence of these effects for the Portuguese aggregate economy (Reis, 2013), our paper shows that these effects can also be present at the local level.

Heterogeneous effects seem to be quite important, quantitatively, when assessing the impact of higher grant eligibility on private firms. This underlines the importance of using data for the entire economy whenever similar studies are conducted. Our contribution is therefore especially relevant given that most recent studies investigating the impact of EU cohesion funds on firm performance, at the firm level, focus exclusively on manufacturing firms (Bachtrögler et al., 2020; Fattorini et al., 2020).

### Robustness

**Fig. 5:**
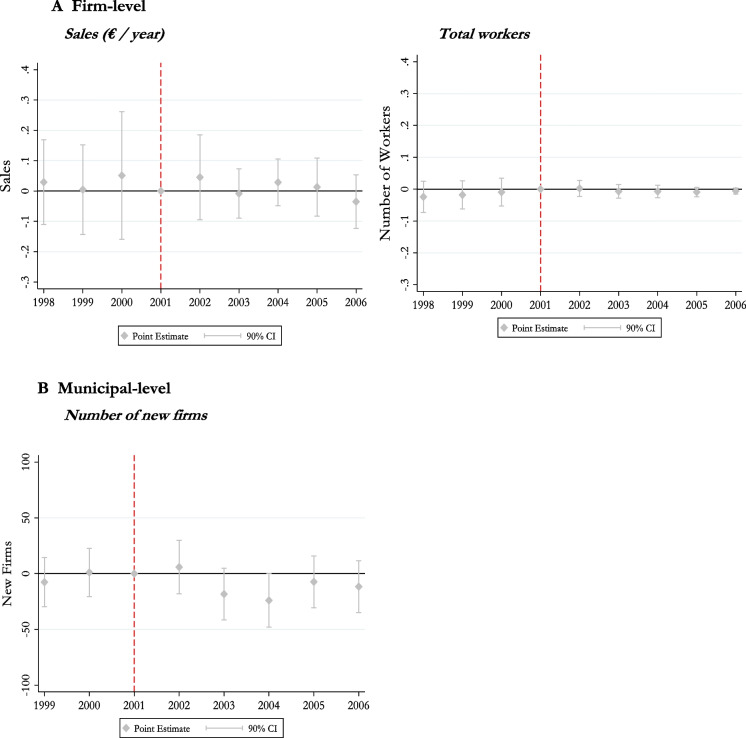
Event study graphs of placebo tests (1998–2006). Notes: This figure presents the results of Eq. (2), analyzing the 1998–2006 period, with a confidence interval of 90%. Sales and total workers were transformed using the inverse hyperbolic sine approach. We define a firm as new if the firm was not observed in the previous year. In 2001, the *Quadros de Pessoal* dataset did not collect information at the worker level. For that reason, we are unable to show the evolution of the *Average wages* in such a timeframe

The diff-in-diff strategy is convincing if it occurs in the presence of no confounding shocks other than the policy (Mayer et al., 2017). The absence of pre-trends, as shown in Fig. 2, is reassuring. Moreover, we take one step further by analyzing possible differential pre-trends for a longer pre-treatment period (since 1998 and until 2006). The placebo tests in Fig. 5A in the Appendix confirm that possible anticipation effects due to the 2002 legislative announcement that reorganized NUTS 3 regions were unlikely to have a confounding effect on our baseline results, as point estimates are close to zero.

However, as there could still exist contemporaneous shocks that may threaten our identification strategy, we subject our evidence to a battery of robustness checks. First, as 2009 and 2010 coincide with one of the greatest recessions in economic history, in the wake of the Sovereign Debt Crisis, there is a concern that Treated municipalities might have been differently affected by shocks during our post-treatment period. If this recession produced differential effects across regions in a way correlated with our breakdown of municipalities into Treated and control, it would introduce confounding effects in our estimates. The event studies in Fig. 2 uncover a sudden drop in the positive effect found in firms’ sales in 2010, the last year of the analysis.^22^ We investigate whether this event is driving our diff-in-diff results by re-estimating Eq. (1) after excluding the years 2009 and 2010 from the sample. We find that these exercises, as reported in Table 11 in the Appendix, are in line with the baseline estimates. Other concurrent episodes that affected the aggregate Portuguese economy include the emergence of China in international trade after 2001 (Cabral et al., 2021) and the EU enlargements of 2004 and 2007 (Caliendo et al., 2021). These events, however, are unlikely to have differential impacts in our treatment and comparison areas. In fact, balance tests provided in Table 9 in the Appendix show that size and sectorial composition of firms in treatment and comparison municipalities are very similar. This is reassuring that shocks other than the one we study in our paper are unlikely to confound our estimates.

A second concern, related with the identification of causal effects from place-based policies, is to construct a valid counterfactual in the absence of the policy. As mentioned in Sect. 3, our control group includes firms from all Portuguese mainland municipalities in NUTS 2 regions close to Treated municipalities whose eligibility status, as well as their neighbors’, remains unchanged. However, as shown in Table 12 in the Appendix, even if we add municipalities in the North NUTS 2 that, on the one hand, are geographic, socioeconomic, and demographically more distant from the Treated area but, on the other hand, also did not experience any change in European funds eligibility status, our results remain unchanged, particularly for the distinct effect on firms’ sales in the Non-Tradable *vis-à-vis* Tradable sectors.

Third, we tested whether our results were robust to a more refined comparison group using matching techniques.^23^ In particular, we use a coarsened exact matching (CEM). The main advantage is the creation of a new control group that resembles the *Treated* firms more closely in terms of pre-treatment observable characteristics—see Appendix B for more details.^24^ This procedure reduces concerns related to confounding effects biasing our estimates, assuming that the more firms are alike in terms of observables before treatment, the more plausible is the parallel trends assumption. In Table 13 in the Appendix, we present estimates combining the CEM and diff-in-diff approaches. Once again, our main results remain significant and mostly unaffected, except for sales and labor productivity in our baseline estimation, whose point estimate becomes statistically non-significant. However, the significant increase in sales for Non-Tradable sectors persists, as well as the positive effect on average wages in every sector. Employment remains statistically indistinguishable from zero.

Fourth, proximity to Lisbon can be a confounding factor if the magnitude of possible spillover effects from the capital to its vicinity changed after the implementation of the new EU eligibility status after 2007. We show a robustness test in Table 14 in the Appendix in which we exclude all firms in the five treated municipalities that are closest to the capital. Our baseline findings remain unchanged, with differences in point estimates across sectors, if anything, becoming more pronounced. The estimated effect on sales, for the entire sample, increases in magnitude but is more noisily measured.

Finally, we show that our results are very similar to baseline if we winsorize data at 5% from each tail, or if we do not winsorize, in Tables 15 and 16 in the Appendix, respectively.

### How do micro and non-micro firms benefit?

In this section, we discuss whether the impacts of increased EU eligibility varied based on firm size to understand how (different types of) firms make decisions. Size is often used as a proxy for a firm’s production and management sophistication (Aw et al., 2011). We divided firms between micro and non-micro (measured in the pre-treatment period and according to the European Commission classification), a decision motivated by the small size of Portuguese private firms (Cabral, 2007).^25^

We further consider three outcome variables: the probability that firms hire at least one top executive manager (using level 1 classification of job levels and the corresponding tasks and skills required by each job level available in *Quadros de Pessoal* dataset), the number of top executive managers, and a measure of the dispersion of wages in the firm (the ratio between the 75 th and the 25 th percentile). These are important variables to shed light on how firms perform as there is plenty of evidence that managerial quality matters for Portuguese firms. Caliendo et al. (2020), for example, show that Portuguese firms respond to shocks by reorganizing their management structure, with considerable impact on their productivity. In addition, Mion and Opromolla (2014) highlight that export experience acquired by managers in previous firms leads their current firm towards higher export performance, and Baptista et al. (2020) find that firms with a higher share of managers and qualified human resources make better investment decisions. We report the results in Table 2.

**Table 2:** Diff-in-diff size analysis

	Sales (ihs)	Total workers (ihs)	Average wages	Labor productivity	Having a top executive	Number of top executives	Wage disparity (P75/P25)
	(1)	(2)	(3)	(4)	(5)	(6)	(7)
Panel C: by size – non-micro versus micro							
Non-micro							
Treated × post-treatment	0.136	0.006	18.957***	2581.234*	0.016**	0.018***	0.015**
	(0.13)	(0.02)	(5.37)	(1398.30)	(0.01)	(0.00)	(0.01)
Adj *R*^2^	0.46	0.81	0.80	0.84	0.61	0.72	0.59
*N*	55,605	55,627	55,627	55,605	55,627	55,627	55,627
Micro							
Treated × post-treatment	0.063*	− 0.005	9.890***	1409.115*	0.001	0.000	0.004
	(0.04)	(0.02)	(2.34)	(673.48)	(0.00)	(0.00)	(0.00)
Adj *R*^2^	0.31	0.75	0.70	0.65	0.54	0.56	0.45
*N*	395,705	395,807	395,807	395,704	395,807	395,807	395,807
Year fixed effects	Yes	Yes	Yes	Yes	Yes	Yes	Yes
Firm fixed effects	Yes	Yes	Yes	Yes	Yes	Yes	Yes

Dependent variables in columns (1) and (2) were transformed using the inverse hyperbolic sine approach. Our regressor of interest, treated × post-treatment, indicates firms producing in one of the 33 treated municipalities during the treatment period (2007–2010). Our analysis includes the 2003–2010 period. Clustered standard errors, at the NUT3 level, are presented in parenthesis. Significance level at which the null hypothesis is rejected: *** 1%, ** 5%, * 10%

We do not find major differences from the baseline estimates for both micro and non-micro firms. The main exception is the lack of statistical significance on sales for non-micro firms, which is a noisy estimate that should be interpreted with caution taking into consideration the relatively large point estimate and the reduction in the number of observations. Importantly, we observe noticeable differences between micro and non-micro firms with respect to the three new outcome variables, suggesting that non-micro firms used the increase in EU eligibility to improve management competence in the extensive and intensive margins. Consequently, wage inequality within these firms increased, and average wages grew at a faster pace in these firms.

### Firm dynamics

Another goal of our study is to identify whether access to a higher eligibility status affected firm dynamics. We address this issue by looking at three outcomes: the total number of firms, number of new firms and probability of exiting the sample.^26^ Table 16 in the Appendix reports our results on the evolution of the total number of firms and the number of new firms at the municipality level, as well as a more granular analysis, at the firm level, analyzing the probability of firms exiting the market, where we employ a linear probability model where the outcome variable takes the value 1 if the firm exits.^27^

In all three cases, the estimated coefficients are statistically indistinguishable from zero, suggesting the absence of eligibility effects on firm dynamics. Importantly, the fact that firm dynamics are not significantly altered in the treatment period suggests that our baseline results are not biased due to composition effects. Indeed, had treatment influenced firms’ entry or exit rates, part of our results could have been driven by a change in the composition of the Treated or the control firms. For example, higher eligibility status could have prevented some below-average firms from leaving the market in Treated municipalities, which in turn could have generated a negative bias on the average performance of firms in such municipalities. This does not seem to be a cause for concern in this case.

Lastly, we show in columns (4) and (5) that our results remain robust to the exclusion of municipalities that were treated by a 2005 reform, the “On the Spot Firm” program (*Empresa na Hora*), which considerably reduced administrative fees, complexity, and time delays for prospective entrepreneurs (Branstetter et al., 2014).^28^

### Are there spillover effects to neighboring municipalities?

We now investigate if there are spillover effects from firms in Treated areas to neighboring untreated areas (Glaeser & Gottlieb, 2009). To that purpose, we redefine our treatment group in this section to include only neighboring municipalities—termed Neighbors, which experienced no change in eligibility, but border a municipality where that change in eligibility occurred. This new treatment group includes firms from 14 different municipalities, as shown in Fig. 1. We keep as control group the same set of firms as in the baseline estimates.

As observed in Table 3, the coefficients of interest for sales and total workers are small and non-statistically significant, suggesting no spillovers. On the contrary, results for average wages and labor productivity confirm the existence of positive spillovers from the Treated areas towards their Neighbors.

**Table 3:** Diff-in-diff spillover results

	Sales (ihs)	Total workers (ihs)	Average wages	Labor productivity
	(1)	(2)	(3)	(4)
Neighbors × post-treatment	0.013	− 0.003	20.147***	2604.540***
	(0.04)	(0.01)	(5.13)	(793.69)
Adj *R*^2^	0.36	0.88	0.74	0.70
*N*	376,606	376,719	376,719	376,605
Year fixed effects	Yes	Yes	Yes	Yes
Firm fixed effects	Yes	Yes	Yes	Yes

Dependent variables in columns (1) and (2) were transformed using the inverse hyperbolic sine approach. Our regressor of interest, neighbors × post-treatment, indicates firms producing in one of the 14 municipalities neighbors to the treated municipalities during the treatment period (2007–2010). Our analysis includes the 2003–2010 period. Clustered standard errors, at the NUT3 level, are presented in parenthesis. Significance level at which the null hypothesis is rejected: *** 1%, ** 5%, * 10%

### Evidence from complementary balance sheet data

Finally, we extend our analysis by using firm-level balance sheet data to explore whether the change in European funds eligibility affected firms’ financial structure. This exercise should be interpreted with caution, given that data is only available since 2004 and comprehensive firm coverage in the SCIE dataset was only achieved in 2006.

We present the results from estimating Eq. (1) in Table 18 in the Appendix. As shown, we do not find statistically significant effects on assets, liabilities, equity, or business profit shares, further suggesting that the reform was not able to structurally change the finance structure of private firms in affected regions.

## Municipal-level results

In this section, we demonstrate that there was a substantial increase in EU funding for firms in the treated area. We then discuss and complement our firm-level findings with further municipal-level administrative data.

In Table 4 column (1), we show how the amount of EU funds directed to firms in the Treated areas increased substantially, in the wake of the eligibility change, relative to the evolution in the control areas. This is expected, given that transfers to firms in these areas were limited before the reform. Relatedly, as presented in Table 4 column (2), transfers from EU funds to local governments, i.e. municipalities, were not impacted. These two pieces of evidence combined confirm that the increase in eligibility was especially relevant for private firms, with no change in funding provided through local authorities.

**Table 4:** Alternative mechanisms

	EU transfers – firms (ihs)	EU transfers – municipalities (ihs)	Government transfers (ihs)	Municipalities’ current expenses (ihs)	Electricity	
					For domestic purposes (ihs)	For industrial purposes (ihs)
	(1)	(2)	(3)	(4)	(5)	(6)
Panel A: treated						
Treated × post-treatment	1.787**	− 0.264	0.015	0.014	0.032***	− 0.016
	(0.72)	(0.76)	(0.01)	(0.02)	(0.00)	(0.10)
Adj *R*^2^	0.43	0.53	0.96	0.97	1.00	0.98
*N*	1096	1096	1096	1096	1096	1096
Panel B: neighbors						
Neighbors × post-treatment	− 0.995	0.141	0.030**	0.005	− 0.010	− 0.073
	(1.28)	(0.22)	(0.01)	(0.08)	(0.01)	(0.08)
Adj *R*^2^	0.446	0.56	0.97	0.79	1.00	0.98
*N*	944	944	944	944	944	944
Year FE	Yes	Yes	Yes	Yes	Yes	Yes
Municipality FE	Yes	Yes	Yes	Yes	Yes	Yes

Our regressors of interest, treated × post-treatment and neighbors × post-treatment, indicate firms producing in treated or neighbors municipalities, respectively during the treatment period (2007–2010). Our analysis spans the 2003–2010 period. Clustered standard errors, at the NUT3 level, are presented in parenthesis. Significance level at which the null hypothesis is rejected: *** 1%, ** 5%, * 10%

We investigate this possibility further by analyzing the volume of transfers from the central government to municipalities and the latter’s current expenditures. If transfers from the central government to Treated municipalities increased sizably in the period following the treatment, our initial results could be due to such change and not the increased eligibility to EU funding itself. In Table 4 column (3), we show evidence that government transfers have not increased in Treated municipalities *vis-à-vis* our control group municipalities.

Additionally, it could also be the case that Treated municipalities autonomously increased their expenditures, financed by higher debt or local taxes, not necessarily due to transfers from the central government. In column (4), we show that Treated municipalities’ current expenditures have not increased, strengthening that there is no evidence that increased spending by the central or local governments played a role in our results.

Veiga (2012), in her study of the determinants of the assignment of EU funds in Portugal, argues that more funds are transferred to municipalities whose electoral results are in line with the party ruling at the national level. We present descriptive evidence that this does not affect our results: in the pre-treatment period (2003–2006), 39% of the municipalities in the Treated group are aligned with the party in the central government, while this figure is 42% for the comparison group. In the post-treatment period (2007–2010), these percentages remain remarkably constant, and the differences are not statistically significant (39% for the Treated, versus 36% for the control).

In Table 4, we analyze whether other municipal level indicators have experienced different growth rates for Treated and non-Treated municipalities. We focus on electricity consumption as a proxy for income. Although electricity for domestic consumption increases by more than 3%, on average, in Treated versus comparison municipalities, we find no effects whatsoever for electricity use by manufacturing. This additional evidence favors the idea that Treated municipalities benefitted from higher income, but access to EU regional funds did not affect firm’s output or productivity in Tradable sectors.

## Conclusion

The EU implements policy initiatives that direct substantial public transfers to lower-income regions across Europe, with the aim of fostering economic convergence. While these policies are designed to stimulate local development, the evidence on their effectiveness remains mixed and often lacks causal empirical grounding (Heinemann et al., 2024). Additionally, there is limited understanding of how public grants affect different industries (Dvouletý et al., 2021).

This paper exploits a quasi-natural experiment resulting from a redistricting decision that led to a discrete expansion in EU grant eligibility for firms in 33 Portuguese municipalities. Using a difference-in-differences framework and administrative microdata, we assess how increased access to cohesion funds relates to firm performance. Our paper adds to a stream of research that empirically assesses the impact of place-based policies, including EU regional policy, on economic development.

We find evidence of a demand-side response: total sales among firms in treated areas rose by over 7% compared to similar firms in unaffected municipalities. Labor productivity also improved. The sales increase is driven entirely by firms in the Non-Tradable, with no effect whatsoever in the more competitive Tradable sectors. Considering firm heterogeneity is crucial to better understand how EU grants impact firms’ performance. Furthermore, increased access to EU funds did not produce a significant increase in employment and only a marginal, though significant, increase in average monthly wages, equivalent to 2% of the average value, with a more pronounced increase for new hires. We also show that the reform had no impact on the financial structure of firms in treated areas. Our results raise questions regarding the effectiveness and sustainability of these effects in the long-term.

Our findings highlight the value of firm-level, sector-specific evaluation of EU funding programs. Leveraging well-defined policy changes can help clarify the nature and mechanisms of these interventions and offer insights into how firms adjust to place-based support.

## Figures and Tables

**Fig. 4:**
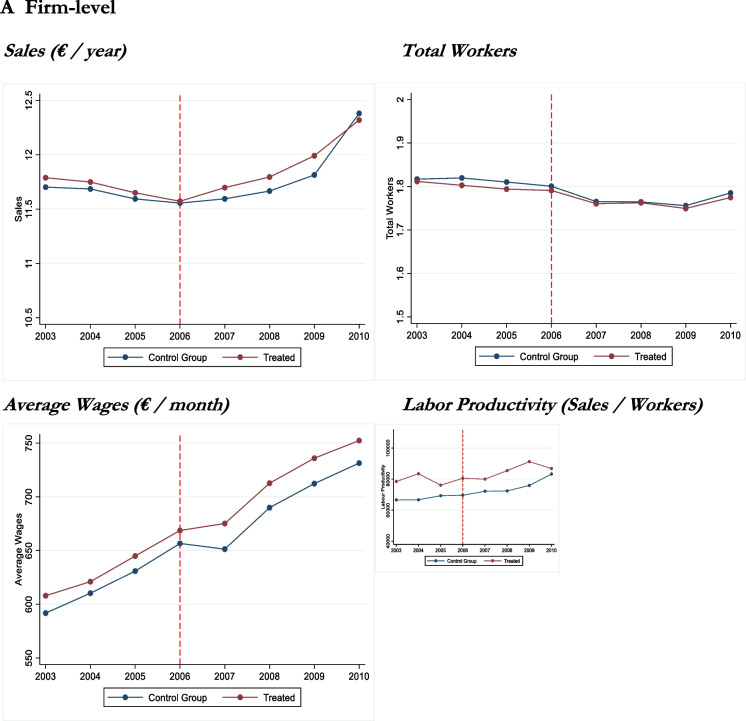
Descriptive graphical evidence. Notes: This figure presents the evolution of our four main outcome variables for the Treated and control group. Sales, total workers, and workers with a bachelor’s degree were transformed using the inverse hyperbolic sine approach

## Data Availability

We rely on two administrative datasets from Statistics Portugal for which access is restricted and the authors do not have the right to republish them. Statistics Portugal, in cooperation with DGEEC, from the Ministry of Education and Science, which has a simple procedure for researchers to access this information in their own computers. More information is available in https://acreditacao.dgeec.medu.pt/. We are happy to help with any requests. The municipal level data will be made available online.

## Appendix 1

Tables 5, 6, 7, 8, 9, 10, 11, 12, 13, 14, 15, 16, 17, and 18

**Table 5:** Data restrictions

Number of observations	Firm-level	% of obs. lost
Treated		
Initial sample	223,922	
Step 1 – Age restriction	222,782	(0.5%)
Step 2 – Minimum wage	178,375	(20.3%)
Step 3 – Sales restriction	164,114	(26.7%)
Step 4 – Single-establishment	158,952	(29.0%)
Control group		
Initial sample	432,668	
Step 1 – Age restriction	430,813	(0.4%)
Step 2 – Minimum wage	344,902	(20.3%)
Step 3 – Sales restriction	320,728	(25.9%)
Step 4 – Single-establishment	310,283	(28.3%)

We imposed four data restrictions. First, we excluded employees whose registered age was under 17 or over 65 years old. Second, we focused solely on workers with a monthly wage higher than the mandatory national minimum wage. Third, we excluded firms without sales in every year they appear in the dataset. Finally, we excluded firms with more than one establishment, as information on sales is not available at that level and given that Portuguese are small in international standards (Cabral, 2007)

**Table 6:** Descriptive statistics – firm-level

Variable	Pre-treatment (2003–2006)				Post-treatment (2007–2010)			
	*N*	Mean	P90	P10	*N*	Mean	P90	P10
Firm-level								
Treated								
Sales (€/year)	75,972	445,023	967,171	11,776	82,940	456,464	974,037	17,343
		(1,057,141)				(1,095,607)		
Total workers	75,972	5.25	11	1	82,980	5.02	10	1
		(9.12)				(8.83)		
Average wages (€/month)	75,972	632.94	924	417	82,980	711.37	1052	450
		(261.12)				(297.35)		
Average wages – new hires	17,066	679.79	1040	421	20,675	768.33	1186	475
		(330.73)				(371.37)		
Average wages – incumbent workers	59,060	653.19	964	425	67,399	737.00	1096	469
		(274.39)				(310.78)		
Labor productivity (sales/workers)	75,972	67,055	151,159	5187	82,940	72,557	159,812	8400
		(100,449)				(104,006)		
Having a top executive	75,972	0.13	1	0	82,980	0.15	1	0
		(0.34)				(0.36)		
Number of top executives	75,972	0.21	1	0	82,980	0.25	1	0
		(0.66)				(0.72)		
Wage disparity (P75/P25)	75,972	1.25	1.71	1.00	82,980	1.24	1.72	1.00
		(0.38)				(0.39)		
Control group								
Sales (€/year)	147,252	417,179	912,261	11,357	162,933	424,518	904,500	15,633
		(1,005,180)				(1,045,329)		
Total workers	147,252	5.47	11.00	1	163,031	5.18	11.00	1.00
		(9.65)				(9.32)		
Average wages (€/month)	147,252	619.55	896.31	414	163,031	688.54	1011.33	450.00
		(259.28)				(289.78)		
Average wages – new hires	30,538	673.07	1035	418	36,037	754.83	1170	461
		(349.66)				(376.92)		
Average wages – incumbent workers	116,318	635.83	927	420	133,799	710.18	1048	455
		(268.87)				(300.48)		
Labor productivity (sales/workers)	147,252	62,072	14,0879	4552	162,932	67,138	149,170	7265
		(91,008)				(95,791)		
Having a top executive	147,252	0.14	1.00	0.00	163,031	0.16	1.00	0.00
		(0.35)				(0.36)		
Number of top executives	147,252	0.23	1.00	0.00	163,031	0.26	1.00	0.00
		(0.72)				(0.76)		
Wage disparity (P75/P25)	147,252	1.24	1.69	1.00	163,031	1.23	1.68	1.00
		(0.38)				(0.37)		
Neighbors								
Sales (€/year)	39,113	474,188	1,095,799	12,877	41,324	505,143	1,160,968	18,662
		(1,072,392)				(1,132,584)		
Total workers	39,113	5.78	13.00	1.00	41,345	5.52	12.00	1.00
		(9.81)				(9.39)		
Average wages (€/month)	39,113	677.24	1029.17	428.00	41,345	765.45	1185.47	464.00
		(294.73)				(335.88)		
Average wages – new hires	9715	744.55	1186	435	10,910	846.53	1338	491
		(383.26)				(433.81)		
Average wages – incumbent workers	30,614	700.80	1076	437	34,381	793.60	1235	475
		(308.42)				(349.52)		
Labor productivity (sales/workers)	39,113	67,030	150,359	5598	41,324	75,216	165,502	9171
		(97,144)				(105,690)		
Having a top executive	39,113	0.18	1.00	0.00	41,345	0.21	1.00	0.00
		(0.38)				(0.41)		
Number of top executives	39,113	0.28	1.00	0.00	41,345	0.33	1.00	0.00
		(0.74)				(0.79)		
Wage disparity (P75/P25)	39,113	1.28	1.79	1.00	41,345	1.27	1.78	1.00
		(0.40)				(0.40)		

**Table 7:** Balance sheet data

Variable	Pre-treatment (2004–2006)		Post-treatment (2007–2010)	
	*N*	Mean	*N*	Mean
Treated				
Assets (ihs)	73,684	12.55	103,390	12.62
		(1.71)		(1.78)
Liabilities (ihs)	73,684	12.03	103,390	12.09
		(2.33)		(2.35)
Equity (ihs)	73,684	7.27	103,390	6.84
		(8.66)		(9.24)
Business profit share	73,186	0.45	102,354	0.48
		(1.84)		(2.01)
Control group				
Assets (ihs)	142,533	12.45	202,116	12.51
		(1.74)		(1.79)
Liabilities (ihs)	142,533	11.87	202,116	11.91
		(2.45)		(2.46)
Equity (ihs)	142,533	7.08	202,116	6.78
		(8.74)		(9.22)
Business profit share	141,509	0.45	200,228	0.48
		(1.95)		(2.1)
Neighbors				
Assets (ihs)	37,772	12.73	52,459	12.80
		(1.68)		(1.74)
Liabilities (ihs)	37,772	12.25	52,459	12.30
		(2.20)		(2.25)
Equity (ihs)	37,772	7.73	52,459	7.41
		(8.42)		(8.98)
Business profit share	37,461	0.45	51,933	0.47
		(1.79)		(1.92)

**Table 8:** Descriptive statistics – municipal-level

Variable	Pre-treatment (2003–2006)				Post-treatment (2007–2010)			
	*N*	Mean	P90	P10	*N*	Mean	P90	P10
Municipal-level								
Treated								
Government transfers	132	6,558,892	12,218,563	3,425,148	132	7,485,156	13,567,579	3,641,778
		(3,166,483)				(4,168,567)		
EU transfers – firms	132	303,156	910,862	0	132	527,547	1,705,995	0
		(635,368)				(953,447)		
EU transfers—municipalities	132	1,454,329	3,213,770	108,071	132	941,022	2,147,947	20,510
		(1,231,352)				(1,058,954)		
Municipalities’ current expenses	132	9411	15,525	3622	132	12,334	19,788	4665
		(5092)				(7250)		
Electricity for domestic purposes	132	30,649	69,150	8152	132	34,508	75,895	9433
		(22,501)				(24,788)		
Electricity for industrial purposes	132	42,720	107,372	4747	132	43,892	116,686	4641
		(43,822)				(44,367)		
Control group								
Government transfers	416	6,631,312	11,113,545	3,657,079	416	7,220,358	11,598,224	3,969,856
		(3,417,540)				(3,682,747)		
EU transfers – firms	416	766,703	2,120,413	0	416	1,299,827	2,789,904	0
		(1,744,032)				(3,998,116)		
EU transfers—municipalities	416	1,207,587	2,582,229	136,479	416	1,118,591	2,398,894	76,561
		(1,429,334)				(1,533,438)		
Municipalities’ current expenses	416	7303	12,947	3159	416	9365	17,289	3943
		(6620)				(8577)		
Electricity for domestic purposes	416	20,544	47,948	4422	416	22,497	51,246	4888
		(27,573)				(29,844)		
Electricity for industrial purposes	416	50,685	103,710	1578	416	52,771	105,731	1669
		(137,873)				(146,005)		
Neighbors								
Government transfers	56	6,941,169	12,352,120	3,834,929	56	7,850,549	14,076,932	4,554,139
		(3,920,103)				(4,566,461)		
EU transfers – firms	56	1,101,135	3,235,845	2482	56	1,568,648	5,336,916	0
		(1,632,942)				(2,991,448)		
EU transfers—municipalities	56	1,024,449	1,954,776	230,069	56	1,144,086	2,390,423	249,216
		(663,896)				(1,104,111)		
Municipalities’ current expenses	56	8240	13,928	2992	56	10,503	17,512	3856
		(7908)				(10,253)		
Electricity for domestic purposes	56	28,299	62,900	4441	56	31,076	66,433	4937
		(39,186)				(43,329)		
Electricity for industrial purposes	56	69,361	290,334	1182	56	71,884	349,284	1364
		(112,850)				(121,779)		

**Table 9:** Balance tests

Variable	Treated	Control group	Diff
	(1)	(2)	(3)
Sales (ihs)	11.57	11.55	0.02
	(3.87)	(3.78)	(0.88)
Total workers (ihs)	1.79	1.80	- 0.01
	(0.93)	(0.95)	(0.84)
Average wages (€/month)	664.15	650.93	13.21
	(273.97)	(274.65)	(0.28)
Average wages – new hires	711.47	710.49	0.98
	(345.47)	(364.68)	(0.94)
Average wages – incumbent workers	683.46	666.74	16.72
	(284.61)	(283.38)	(0.23)
Labor productivity (sales/workers)	67,683.97	63,256.22	4427.75
	(100,658.07)	(93,009.20)	(0.14)
Having a top executive	0.14	0.14	− 0.00
	(0.35)	(0.35)	(0.847)
Number of top executives	0.23	0.24	− 0.02
	(0.69)	(0.74)	(0.61)
Wage disparity (P75/P25)	1.25	1.24	0.01
	(0.39)	(0.39)	(0.60)
% of firms in tradable sector	0.34	0.35	− 0.01
	(0.48)	(0.48)	(0.76)
% of micro firms	0.87	0.87	0.00
	(0.33)	(0.33)	(0.97)
% of firms in manufacturing sector	0.33	0.32	0.01
	(0.47)	(0.47)	(0.71)
% of firms in services sector	0.60	0.61	− 0.01
	(0.49)	(0.49)	(0.72)
*N*	19,826	38,300	58,126

The analysis corresponds to 2006, the last year prior to treatment. Clustered standard errors, at the NUT3 level, are presented in parenthesis, except for column (3), where *p*-values are in parenthesis; significance level at which the null hypothesis is rejected

**Table 10:** Robustness: employing a logarithmic transformation

	Sales (log)	Total workers (log)
	(1)	(2)
Panel A: full sample		
Treated × post-treatment	0.071*	− 0.003
	(0.04)	(0.02)
Adj *R*^2^	0.37	0.89
*N*	451,318	451,442
Panel B: by sector – tradable versus non-tradable		
Non-tradable		
Treated × post-treatment	0.091**	− 0.003
	(0.04)	(0.02)
Adj *R*^2^	0.37	0.88
*N*	297,737	297,811
Tradable		
Treated × post-treatment	0.021	− 0.001
	(0.06)	(0.02)
Adj *R*^2^	0.39	0.91
*N*	151,226	151,274
Year fixed effects	Yes	Yes
Firm fixed effects	Yes	Yes

Dependent variables suffered a logarithmic transformation; our regressor of interest, treated × post-treatment, indicates firms producing in one of the 33 Treated municipalities during the treatment period (2007–2010). Our analysis includes the 2003–2010 period. Clustered standard errors, at the NUT3 level, are presented in parenthesis; significance level at which the null hypothesis is rejected: ** 5%, * 10%

**Table 11:** Robustness: without crisis period (2003–2008)

	Sales (ihs)	Total workers (ihs)	Average wages	Labor productivity
	(1)	(2)	(3)	(4)
Panel A: baseline				
Treated × post-treatment	0.102*	0.002	10.523***	1653.125**
	(0.06)	(0.02)	(2.28)	(762.81)
Adj *R*^2^	0.41	0.89	0.73	0.73
*N*	335,063	335,063	335,063	335,063
Panel B: by sector – tradable versus non-tradable				
Non-tradable				
Treated × post-treatment	0.107*	0.003	10.657***	1913.341*
	(0.06)	(0.02)	(2.78)	(949.51)
Adj *R*^2^	0.41	0.88	0.73	0.76
*N*	220,039	220,039	220,039	220,039
Tradable				
Treated × post-treatment	0.073	− 0.000	11.487***	443.604
	(0.08)	(0.02)	(3.34)	(752.74)
Adj R2	0.42	0.91	0.74	0.67
N	112,686	112,686	112,686	112,686
Year fixed effects	Yes	Yes	Yes	Yes
Firm fixed effects	Yes	Yes	Yes	Yes

Dependent variables in columns (1) and (2) were transformed using the inverse hyperbolic sine approach; our regressor of interest, treated × post-treatment, indicates firms producing in one of the 33 Treated municipalities during the treatment period (2007–2010). Our analysis includes the 2003–2008 period. Clustered standard errors, at the NUT3 level, are presented in parenthesis; significance level at which the null hypothesis is rejected: *** 1%, ** 5%, * 10%

**Table 12:** Robustness: including north region in the control group

	Sales (ihs)	Total workers (ihs)	Average wages	Labor productivity
	(1)	(2)	(3)	(4)
Panel A: baseline				
Treated × post-treatment	0.036	− 0.017	11.947***	1337.932***
	(0.03)	(0.02)	(1.92)	(350.33)
Adj *R*^2^	0.36	0.88	0.75	0.71
*N*	1,094,724	1,094,982	1,094,982	1,094,716
Panel B: by sector – tradable versus non-tradable				
Non-tradable				
Treated × post-treatment	0.070*	− 0.022	11.705***	996.115*
	(0.04)	(0.02)	(2.17)	(482.56)
Adj *R*^2^	0.36	0.86	0.75	0.73
*N*	703,766	703,933	703,933	703,759
Tradable				
Treated × post-treatment	− 0.048	− 0.010	12.648***	1280.735*
	(0.04)	(0.02)	(3.08)	(697.29)
Adj *R*^2^	0.38	0.90	0.75	0.65
*N*	384,954	385,043	385,043	384,953
Year fixed effects	Yes	Yes	Yes	Yes
Firm fixed effects	Yes	Yes	Yes	Yes

Dependent variables in columns (1) and (2) were transformed using the inverse hyperbolic sine approach; our regressor of interest, treated × post-treatment, indicates firms producing in one of the 33 Treated municipalities during the treatment period (2007–2010). Our analysis includes the 2003–2010 period. Our control group includes the north region (see Fig. 1). Clustered standard errors, at the NUT3 level, are presented in parenthesis; significance level at which the null hypothesis is rejected: *** 1%, * 10%

**Table 13:** Robustness: coarsened exact matching

	Sales (ihs)	Total workers (ihs)	Average wages	Labor productivity
	(1)	(2)	(3)	(4)
Panel A: baseline				
Treated × post-treatment	0.058	− 0.002	11.071***	1154.560
	(0.04)	(0.02)	(2.25)	(826.46)
Adj *R*^2^	0.38	0.89	0.74	0.73
*N*	298,555	298,634	298,634	298,554
Panel B: by sector – non-tradable versus tradable				
Non-tradable				
Treated × post-treatment	0.088**	− 0.003	11.101***	1959.416*
	(0.04)	(0.02)	(2.73)	(1027.61)
Adj *R*^2^	0.38	0.87	0.74	0.75
*N*	198,849	198,895	198,895	198,848
Tradable				
Treated × post-treatment	− 0.006	0.002	11.047***	− 463.228
	(0.06)	(0.02)	(2.72)	(790.16)
Adj *R*^2^	0.38	0.90	0.73	0.66
*N*	99,706	99,739	99,739	99,706
Year fixed effects	Yes	Yes	Yes	Yes
Firm fixed effects	Yes	Yes	Yes	Yes

Dependent variables in columns (1) and (2) were transformed using the inverse hyperbolic sine approach; our regressor of interest, treated × post-treatment, indicates firms producing in one of the 33 Treated municipalities during the treatment period (2007–2010). Our analysis includes the 2003–2010 period. Clustered standard errors, at the NUT3 level, are presented in parenthesis; significance level at which the null hypothesis is rejected: *** 1%, ** 5%, * 10%

**Table 14:** Robustness: excluding the five closest municipalities to Lisbon

	Sales (ihs)	Total workers (ihs)	Average wages	Labor productivity
	(1)	(2)	(3)	(4)
Panel A: baseline				
Treated × post-treatment	0.091	− 0.009	10.941***	1455.967*
	(0.06)	(0.02)	(2.30)	(707.41)
Adj *R*^2^	0.36	0.88	0.73	0.71
*N*	417,949	418,069	418,069	417,948
Panel B: by sector – non-tradable versus tradable				
Non-tradable				
Treated × post-treatment	0.116*	− 0.009	11.181***	1682.024*
	(0.06)	(0.02)	(3.45)	(815.07)
Adj *R*^2^	0.36	0.87	0.73	0.74
*N*	275,910	275,982	275,982	275,909
Tradable				
Treated × post-treatment	0.027	− 0.008	9.979***	282.790
	(0.07)	(0.02)	(2.90)	(937.14)
Adj *R*^2^	0.37	0.90	0.73	0.64
*N*	139,851	139,897	139,897	139,851
Year fixed effects	Yes	Yes	Yes	Yes
Firm fixed effects	Yes	Yes	Yes	Yes

Dependent variables in columns (1) and (2) were transformed using the inverse hyperbolic sine approach; our regressor of interest, treated × post-treatment, indicates firms producing in one of the 33 Treated municipalities during the treatment period (2007–2010), with the exception of firms in one of the five closest municipalities to Lisbon (Arruda dos Vinhos, Sobral de Monte Agraço, Benavente, Alenquer, Torres Vedras. Our analysis includes the 2003–2010 period. Clustered standard errors, at the NUT3 level, are presented in parenthesis; significance level at which the null hypothesis is rejected: *** 1%, * 10%

**Table 15:** Robustness: winsorize 95%

	Sales (ihs)	Total workers (ihs)	Average wages	Labor productivity
	(1)	(2)	(3)	(4)
Panel A: baseline				
Treated × post-treatment	0.073*	− 0.003	9.988***	1054.536*
	(0.04)	(0.02)	(1.93)	(516.42)
Adj *R*^2^	0.35	0.86	0.75	0.74
*N*	451,318	451,442	451,442	451,317
Panel B: by sector – non-tradable versus tradable				
Non-tradable				
Treated × post-treatment	0.092**	− 0.004	10.446***	1444.680**
	(0.04)	(0.02)	(2.70)	(556.93)
Adj *R*^2^	0.35	0.86	0.75	0.77
*N*	297,737	297,811	297,811	297,736
Tradable				
Treated × post-treatment	0.024	− 0.001	8.965***	96.152
	(0.06)	(0.02)	(1.85)	(736.24)
Adj *R*^2^	0.36	0.88	0.75	0.68
*N*	151,226	151,274	151,274	151,226
Year fixed effects	Yes	Yes	Yes	Yes
Firm fixed effects	Yes	Yes	Yes	Yes

Dependent variables in columns (1) and (2) were transformed using the inverse hyperbolic sine approach; our regressor of interest, treated × post-treatment, indicates firms producing in one of the 33 Treated municipalities during the treatment period (2007–2010). Our analysis includes the 2003–2010 period. Clustered standard errors, at the NUT3 level, are presented in parenthesis; significance level at which the null hypothesis is rejected: *** 1%, ** 5%, * 10%

**Table 16:** Robustness: without winsorize

	Sales (ihs)	Total workers (ihs)	Average wages	Labor productivity
	(1)	(2)	(3)	(4)
Panel A: baseline				
Treated × post-treatment	0.074*	− 0.004	12.132***	3276.760
	(0.04)	(0.02)	(3.09)	(4186.72)
Adj *R*^2^	0.36	0.88	0.53	0.45
*N*	451,318	451,442	451,442	451,317
Panel B: by sector – non-tradable versus tradable				
Non-tradable				
Treated × post-treatment	0.095**	− 0.004	12.729***	5067.133
	(0.04)	(0.02)	(3.62)	(6503.62)
Adj *R*^2^	0.36	0.87	0.52	0.45
*N*	297,737	297,811	297,811	297,736
Tradable				
Treated × post-treatment	0.020	− 0.003	10.838**	− 2161.594
	(0.06)	(0.02)	(4.69)	(1253.27)
Adj *R*^2^	0.38	0.90	0.55	0.33
*N*	151,226	151,274	151,274	151,226
Year fixed effects	Yes	Yes	Yes	Yes
Firm fixed effects	Yes	Yes	Yes	Yes

Dependent variables in columns (1) and (2) were transformed using the inverse hyperbolic sine approach; our regressor of interest, treated × post-treatment, indicates firms producing in one of the 33 Treated municipalities during the treatment period (2007–2010). Our analysis includes the 2003–2010 period. Clustered standard errors, at the NUT3 level, are presented in parenthesis; significance level at which the null hypothesis is rejected: *** 1%, ** 5%, * 10%

**Table 17:** Firm dynamics

	Number of firms (ihs)	Number of new firms (ihs)	Probability of closing	Number of firms (ihs)	Number of new firms (ihs)
Sample:	Entire sample			Without *on the stop firm*	
	(1)	(2)	(3)	(4)	(5)
Treated × post-treatment	− 0.011	0.046	0.003	− 0.013	0.057
	(0.04)	(0.05)	(0.01)	(0.04)	(0.05)
Adj *R*^2^	0.99	0.91	0.35	0.99	0.90
*N*	1096	1096	451,442	963	963
Year fixed effects	Yes	Yes	Yes	Yes	Yes
Municipal fixed effects	Yes	Yes	Yes	Yes	Yes
Firm fixed effects	No	No	Yes	No	No

Dependent variables in columns (1) and (2) have suffered an inverse hyperbolic sine transformation. The first two columns are presented at the municipality level, while column (3) is at the firm level. We define entry in the market if the firm was not observed in the previous 2 years and exit if the firm is not observed in the following 2 years. Our regressor of interest, treated × post-treatment, indicates firms producing in one of the 33 Treated municipalities during the treatment period (2007–2010). In columns (4) and (5), we do not include the municipalities, both in Treated and control group, that participated in *Empresa na Hora* (*On the Spot*) program after the treatment was implemented (Branstetter et al., 2014). These include Abrantes, Alcobaça, Bombarral, Caldas da Rainha, Óbidos, Santarém, Tomar, Torres Vedras, Águeda, Aljustrel, Almeida, Aveiro, Beja, Cantanhede, Castelo Branco, Coimbra, Covilhã, Elvas, Estremoz, Évora, Figueira da Foz, Figueira de Castelo Rodrigo, Fornos de Algodres, Guarda, Idanha-a-Nova, Ílhavo, Moura, Oliveira do Bairro, Ovar, Portalegre, Santigado do Cacém, Seia, Tondela, and Viseu. Our analysis includes the 2003–2010 period. Clustered standard errors, at the NUT3 level, are presented in parenthesis; significance level at which the null hypothesis is rejected

**Table 18:** Diff-in-diff balance sheet results

	Assets (ihs)	Liabilities (ihs)	Equity (ihs)	Business profit share
	(1)	(2)	(3)	(4)
Treated × post-treatment	− 0.006	0.002	− 0.110	0.017
	(0.02)	(0.02)	(0.08)	(0.02)
Adj *R*^2^	0.86	0.74	0.66	0.12
*N*	427,776	427,776	427,776	423,685
Year fixed effects	Yes	Yes	Yes	Yes
Firm fixed effects	Yes	Yes	Yes	Yes

Dependent variables in columns (1), (2), and (3) were transformed using the inverse hyperbolic sine approach. Our regressor of interest, treated × post-treatment, indicates firms producing in one of the 33 Treated municipalities during the treatment period (2007–2010). Our analysis only comprises the 2004–2010 period due to data coverage in SCIE dataset. Business profit share is defined as gross operating surplus as a share of the gross value added (at market prices). Clustered standard errors, at the NUT3 level, are presented in parenthesis; significance level at which the null hypothesis is rejected

Figs. 3, 4, and 5

## Appendix 2. Further details on the CEM method

The first phase of the CEM method is to stratify firms according to their observables. In our case, we form groups of firms that are in the same decile regarding the distribution of sales, number of workers, and average wages in the year preceding treatment (i.e., 2006). This way, we create a total of 1000 stratums so that firms in the same stratum belong to the same decile in the distribution of sales, number of workers, and average wages. Out of those 1000 stratums, in only 12 are firms in both the Treated and control groups, so firms in the remaining stratums were excluded from this analysis for not having a compatible enough counterfactual. From our initial baseline specification, about a third of the observations were excluded—taking us from around 451,000 to 297,000 observations.

The second part of the method is to estimate our DiD equation on this new reduced sample, with the CEM weights. The CEM weights guarantee that within each stratum, the sum of the weights of Treated and control group firms are the same and that each Treated observation is weighted the same, regardless of its stratum.

## Footnotes

1 NUTS (Nomenclature of territorial units for statistics) classification is set up by the Eurostat to divide EU’s territory and produce regional statistics.

2 From 2013 to 2016, for example, there were substantial recoding changes in countries such as France, Poland, Ireland, Germany, and Finland: https://ec.europa.eu/eurostat/documents/345175/17782619/2018-20-NUTS3-NUTS-change-2013-to-2016-EU28.pdf/66158fd2-f57d-8a16-bb6a-2551b8b7b5e9?t=1698689445182. In fact, the last update was made on March 6, 2024. For a comprehensive list of all changes, see https://ec.europa.eu/eurostat/web/nuts/history.

3 An exception is Becker et al. (2018), who compare the effects of gaining versus losing eligibility status.

4 Other empirical approaches have been attempted such as instrumental variable estimates—Ramajo et al. (2008); (dynamic) panel data techniques—Rodríguez-Pose and Fratesi (2004); a combination of the two—Bouayad-Agha et al. (2013); Bayesian methods – Cuaresma et al. (2014); or spatial growth models – Fiaschi et al. (2018). However, the empirical evidence is mixed and remains controversial (Dall’erba and Fang, 2015).

5 Pellegrini et al. (2013) confirm the results of Becker et al. (2010) using Eurostat data.

6 Gobillon et al. (2012) and Mayer et al. (2017) discuss similar schemes in France, while Einiö and Overman (2020) and Criscuolo et al. (2019) do it for two cases in the UK using firm-level data. Examining manufacturing firms in Italy, Bernini and Pellegrini (2011) investigated the impact of state regional policy to subsidized firms, finding higher growth rate of sales, employment, and investments, although with a negative impact on total factor productivity.

7 We show in Sect. 4.3 that possible anticipation effects are unlikely to confound our effects.

8 As can be seen in Fig. 3 in the Appendix, the previous NUTS 2 region of Lisbon and the Tagus valley was severely reduced with the incorporation of some of its NUTS 3 regions in other NUTS 2: Oeste and Médio Tejo was transferred to NUTS 2 Centro, whereas Lezíria do Tejo was transferred to NUTS 2 Alentejo.

9 Cases of self-employment are excluded. In addition, organizations falling outside the partnership or sole proprietorship legal definitions were also omitted due to their non-profit nature.

10 We imposed four data restrictions. First, we excluded employees whose registered age was under 17 or over 65 years old. Second, we focused solely on workers with a monthly wage higher than the mandatory national minimum wage. Third, we excluded firms without sales in every year they appear in the dataset. Finally, we excluded firms with more than one establishment, as information on sales is not available at that level and given that Portuguese are small in international standards (Cabral 2007). We quantify the impact of these restrictions in the Table 5 in the Appendix.

11 For instance, Carreira and Paulino (2016) find that the crisis had negative impacts not only on zombie firms but also on high-productivity firms due to credit market distortions. Some policy responses also had different regional effects. Audretsch et al. (2020) and Branco et al. (2023), for example, show that the introduction of tolls in a subset of Portuguese highways covering about 1/3 of the grid in 2011 reduced turnover and productivity in treated firms vis-à-vis firms more distant to the affected infrastructure.

12 Our results are robust if we do not winsorize our sample, or winsorize at different values, as discussed in Sect. 4.3.

13 We apply the same data restrictions described in footnote 10. This dataset has been used, *inter alia*, to study how the organization of management production impacts revenues and productivity (Caliendo et al., 2020) and the effects of the rise of transportation costs in some highways during the Great Recession (Branco et al., 2023).

14 Unlike what happens in some EU member states, including Belgium and the Netherlands, NUTS 2 regions do not inherit political power, and their administrative competencies are extremely limited. This was the only change since NUTS 2 were defined in 1989 until the end of our sample period. In 2023, a new change in NUTS 2 was approved for Portugal, with effect after 2028. In Portugal, there is no layer of government between the central government and municipalities.

15 Our results are robust to the inclusion of the municipality fixed effects and are shown in previous working paper versions of this paper available on-line.

16 Figure 4 in the Appendix presents descriptive graphical evidence that further corroborates the plausibility of the parallel trend assumption, for all outcome variables, in this context (Angrist and Pischke, 2008).

17 We also find an effect above 7% if we use the logarithmic instead of the inverse hyperbolic sine transformation. We present these results in Table 10 in the Appendix.

18 Bernini and Pelligrini (2011) study Italian public subsidies and show that output growth is 8–10% higher in subsidized *vis-à-vis* non-subsidized firms, while Cerqua and Pellegrini (2014), in a similar context, uncover a 6.5–8% higher yearly growth rate of turnover in subsidized firms. Decramer and Vanormelingen (2016) investigate an investment subsidy program in Flanders and estimate an increase in sales growth rate for firms up to 45 employees, and in particular for firms with one employee. Srhoj et al. (2021) analyze a public subsidy in Croatia for SMEs and find an average increase in sales of 5.2%, more pronounced in firms with less than 20 workers.

19 Shroj et al. (2016) examine a Croatian public subsidy for SMEs, while Benkovskis et al. (2019) study the impact of EU regional support on Latvian firms and find positive effects on average wages, uncovering effects of 6.5% and 5–8%, respectively.

20 Bondonio and Greenbaum (2014) analyze the effect of the European Regional Development Fund on firms in the Piemonte region, while Banai et al. (2017) examine the impact of EU Cohesion Policy funds on Hungarian firms, and both estimate positive effects on the number of employees per firm.

21 The Tradable sector was defined according to the statistical classification of economic activities in the European Community and covers agriculture, forestry and fishing; mining and quarrying; manufacturing; transportation and storage; professional, scientific and technical activities; and administrative and support service activities.

22 Possible explanations might be in line with Becker et al. (2018) who show that eligibility effects weakened during the financial crisis, and with Fantino and Cannone (2014) who find that the impact of policies designed to boost innovation in small firms in an Italian region was effective exclusively in the short-run.

23 Belluci et al. (2019), for example, use propensity score matching methods to study the effects of two public R&D subsidy programs in Italy.

24 Iacus et al. (2012) show that this method produces lower model dependence, estimation error, variance, bias, and reduces imbalance *vis-à-vis* remaining commonly employed matching methods.

25 The EC defines micro-enterprises as those employing fewer than 10 persons and whose annual turnover does not exceed EUR 2 million (for more information, https://single-market-economy.ec.europa.eu/smes/sme-fundamentals/sme-definition_en).

26 Figueiredo et al. (2002) and Carias et al. (2022) show that Portuguese entrepreneurs tend to locate their businesses in their place of residence. Furthermore, Branco et al. (2023) report that they do not tend to move in response to negative shocks.

27 For an extended revision of the literature on firm exit, see Cefis et al. (2022).

28 Due to resource constraints, the one-stop shops were first piloted in four municipalities and extended later to other regions.

## Code availability

We want to assure that the code that is needed to fully replicate all our results will be made available on-line if the paper is accepted for publication.
